# The Time Window for Generation of Dendritic Spikes by Coincidence of Action Potentials and EPSPs is Layer Specific in Somatosensory Cortex

**DOI:** 10.1371/journal.pone.0033146

**Published:** 2012-03-13

**Authors:** Debora Ledergerber, Matthew Evan Larkum

**Affiliations:** 1 Kavli Institute for Systems Neuroscience, Norwegian University of Science and Technology, Trondheim, Norway; 2 Physiologisches Institut, Universität Bern, Bern, Switzerland; 3 Neuroscience Research Center – Campus Mitte, Charité Universitätsmedizin Berlin, Berlin, Germany; University of Cincinnatti, United States of America

## Abstract

The precise timing of events in the brain has consequences for intracellular processes, synaptic plasticity, integration and network behaviour. Pyramidal neurons, the most widespread excitatory neuron of the neocortex have multiple spike initiation zones, which interact via dendritic and somatic spikes actively propagating in all directions within the dendritic tree. For these neurons, therefore, both the location and timing of synaptic inputs are critical. The time window for which the backpropagating action potential can influence dendritic spike generation has been extensively studied in layer 5 neocortical pyramidal neurons of rat somatosensory cortex. Here, we re-examine this coincidence detection window for pyramidal cell types across the rat somatosensory cortex in layers 2/3, 5 and 6. We find that the time-window for optimal interaction is widest and shifted in layer 5 pyramidal neurons relative to cells in layers 6 and 2/3. Inputs arriving at the same time and locations will therefore differentially affect spike-timing dependent processes in the different classes of pyramidal neurons.

## Introduction

Timing is a central concept in cortical function. At the network level, information is encoded in the spiking of neurons and there is much debate about the level of precision that is important [1], [2]. At the cellular level important processes have been hypothesized to be dependent on the timing of input and output such as spike-timing dependent plasticity “STDP” [3]. The notion of timing is particularly important in pyramidal neurons, the principle excitatory neurons of the neocortex. With their elongated dendritic trees spanning several cortical layers they can independently process different classes of synaptic input within the same neuron [4]. The synaptic inputs that can contribute to the input/output function for each pyramidal neuronal type is determined by the specific layers spanned by their dendritic trees and the laminar profile of activity throughout the cortex which is specific to each pyramidal cell class.

Recently it has become clear that the input/output function of pyramidal neurons is also profoundly influenced by the computational properties of the dendritic tree itself [5], [6], [7]. The dendrites of all cortical pyramidal neurons have been shown to have Na^+^, K^+^ and Ca^2+^ channels [8], [9], [10] that contribute to the active propagation of signals and to the generation of local spikes [11], [12], [13], [14], [15]. The final output from the pyramidal neurons is the generation of action potentials in the axon initial segment [16], [17], but the computational power of the pyramidal neuron is greatly enhanced by the interaction of these APs with the sub-regions of the dendritic tree that generate local spikes [5], [18].

Neocortical pyramidal neurons have a spike initiation zone in the apical dendrite [10], [11], [12], [14], [19]. The dendritic spike generated in this location is composed of an initial fast component that has been shown to be mediated by voltage-sensitive Na^+^ channels followed by a slower Ca^2+^-dependent component [10], [20]. In L5 pyramidal neurons the 2^nd^ component is particularly pronounced and typically drives the soma to fire a burst of APs [21], [22], [23]. In L2/3 and L6 neurons, the 2^nd^ component contributes to further somatic depolarization but does not necessarily trigger axonal firing. The dendritic and axonal spike initiation zones are coupled by the influence of the backpropagating action potential (bAP) that lowers the threshold for the initiation of the dendritic spike. This phenomenon, known as “backpropagation activated calcium spike firing” (BAC firing) [18] is strongly dependent on the relative timing of input to the proximal and distal initiation zones. The generation of a dendritic spike under these circumstances represents a mechanism for pyramidal neurons to detect the coincidence of proximal and distal input to the dendritic tree.

In this paper, we investigated the time window of coincidence detection in L2/3, L5 and L6 pyramidal neurons of the somatosensory cortex in rats using simultaneous dual patch-clamp recordings from the cell body and apical dendrite and we show that all three types of pyramidal neurons have a specific time window for somato-dendritic spike interaction.

## Materials and Methods

The study was approved by the Veterinary office of the Canton Bern, Switzerland, permission number 90/08.

### Slice preparation

Experiments were performed in somatosensory neocortical slices from postnatal day 28–49 Wistar rats (n = 26) using procedures described previously [9]. Briefly, rats were decapitated and the brain was quickly removed into cold (0–4°C), oxygenated physiological solution containing the following (in mM): 125 NaCl, 2.5 KCl, 1.25 NaH_2_PO_4_, 25 NaHCO_3_, 1 MgCl_2_, 2 CaCl_2_, and 25 glucose; pH 7.4. Parasagittal slices, 300 µm thick, were cut from the tissue block with a vibratome (Microm) and kept at 37°C for 30 min and then at room temperature until use.

### Electrophysiology

All experiments were performed at 32.0±0.5°C. Single pyramidal neurons were identified using infrared Dodt gradient contrast or oblique illumination and a CCD camera (CoolSnap ES, Roper Scientific). Slices were perfused with the same extracellular solution mentioned above. Recording pipettes were filled with intracellular solution containing the following: 130 mM K-gluconate, 5 mM KCl, 30 mM HEPES, 10 mM Phospho-kreatine, 4 mM MgATP, and 0.3 mM GTP; pH 7.3. The somatic pipette contained in addition 10–50 µM Alexa 594 (Invitrogen), 100 µM Oregon Green BAPTA-1 (OGB-1, Invitrogen), and 0.2% Biocytin (Sigma). Dual whole-cell voltage recordings were performed from the soma and dendrites (6–10 and 20–40 MΩ pipette resistances respectively) using Axoclamp 2A (Axon Instruments) and Dagan BVC-700A amplifiers (Dagan Corporation). Data were acquired with an ITC-18 board (Instrutech) and custom software written for the Igor environment (Wavemetrics). After recordings, slices were fixed and stained as described previously [14] for later reconstruction of the investigated neurons. Data analysis was performed using Igor software (Wavemetrics) and Excel (Microsoft).

The dendritic recording was made at least 20 min after establishing the somatic recording to allow intracellular spread of the dyes from the soma. Dendrites were targeted with infrared-scanning gradient contrast (IR-SGC) [24] or an overlay of the separately acquired epifluorescence image with an obliquely illuminated IR image using custom software. We used a Leica TCS SP2 confocal scanner or an Olympus BX-51WI microscope with a 60X objective. Dendritic spikes were elicited with direct dendritic current injection by a pipette placed in the spike initiation zone [10], [19], [23]. The regenerative component of the dendritic AP was calculated by subtracting the predicted non-regenerative component (using the previous sub-threshold traces) from the suprathreshold dendritic recording [10], [19]. We found no evidence that the precise location of current injection (inside the initiation zone) alters the timing of coincidence detection.

### Statistics

**Figure 1:**
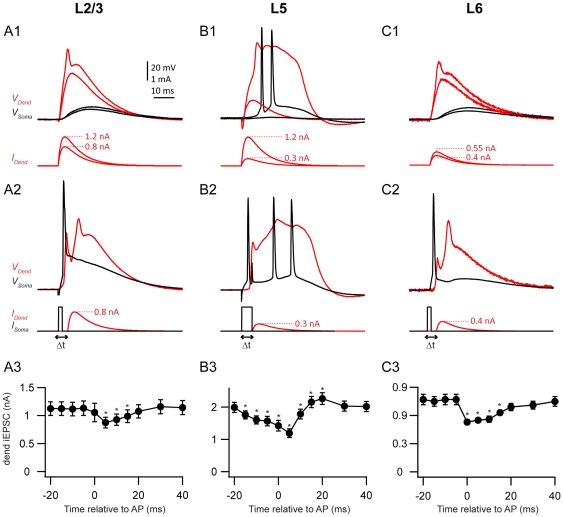
Somato-dendritic coupling for pyramidal neurons in different layers of the neocortex. Cell types are arranged in columns (A, L2/3; B, L5; C, L6). Row 1) Injection of EPSC-waveform current (lower panels) into the apical dendrite below and above threshold for the generation of a dendritic spike (red traces) which propagated to the soma (black traces). Row 2) Sub-threshold current injection from row 1 5 ms after an axonal AP elicited by somatic current injection (black traces in bottom panels). Row 3) Average threshold current at the dendritic electrode for the generation of a dendritic spike in the presence of a backpropagating AP for various time intervals (Δt). Values are presented as mean with standard error. Asterisks indicate significant deviation from baseline (threshold determined in row 1) tested with the Holm-Šidák.

**Figure 2:**
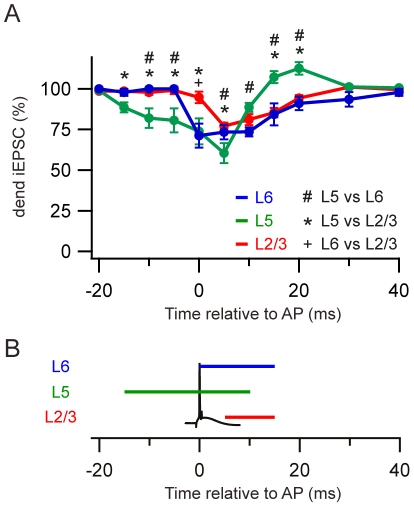
Time windows for AP/EPSP coincidence detection. A) Average normalized dendritic spike thresholds for L6 (blue), L5 (green) and L2/3 (red) pyramidal neurons for different dendritic versus somatic times. Statistical difference is indicated for comparisons between cell types (#, L5 vs. L6; *, L5 vs. L2/3; +, L6 vs. L2/3) using Holm-Šidák (p<0.05). B) Time windows for coincidence detection showing intervals where the threshold was significantly lower than baseline (Holm-Šidák).

All statistics were calculated using commercial software (SigmaStat, Systat Software Inc.; San Jose, CA). If not otherwise indicated values represent means ± s.e.m. All data were tested for normality and equal variance. Statistical comparisons of spike thresholds were performed using 2-way repeated measurement ANOVA to test for effects of time versus baseline (Fig. 1) or for time versus cell type (Fig. 2). A significance level of 5% was chosen.

## Results

The aim of this study was to investigate the coupling of the tuft dendrite with the cell body across the pyramidal cell classes of the cortex. The coupling was assessed in terms of the coincidence time window during which a backpropagating AP influenced the threshold for the generation of a dendritic spike. We carried out dual whole-cell patch clamp recordings from the dendrites and somata in layers 6, 5 b (thick-tufted cells) & 2/3 in the somatosensory neocortex of rats (see Table 1 for detailed experimental parameters). A transient current resembling a compound EPSC (EPSC_inj_) was injected into the dendrite (Fig. 1, upper panels; for further details see Methods). Axonal APs were evoked with 2-ms somatic current injection just above the AP threshold (Fig. 1B, middle panels).

**Table 1:** Experimental parameters and cell properties across pyramidal cell types.

	L2/3	L5	L6
**Soma location, distance from pia (µm)**	582±50	1093±111	1548±65
**Dendritic patch location, distance from soma (µm)**	238±45	699±102	399±52
**Baseline threshold for dendritic spike (pA)**	1142±419	2011±553	770±192
**Threshold for dendritic spike combined with AP (pA)**	858±350	1144±480	500±100
**Average age of recorded rats (days post natal)**	31±3	41±8	29±1
**n**	12	9	5

Values are given as means with standard deviations.

We first determined the threshold for a dendritic spike using only dendritic current injection (Fig. 1, upper panels). The threshold for dendritic spikes was lowest in L6 pyramidal neurons (avg 770±192 pA, n = 5; Fig. 1C1; Table 1), highest in L5 neurons (avg 2011±553 pA, n = 9; Fig. 1B1) and intermediate in L2/3 neurons (avg 1142±419 pA, n = 12; Fig. 1A1). However, this threshold decreased when the cell fired an axonal AP 5 ms before the dendritic spike (Fig. 1, middle panels; Table 1). We assessed the reduction in threshold for time intervals (Δt) between −20 and 40 ms (Fig. 1, lower panels). One way repeated measures ANOVA for each group of pyramidal neurons revealed that there was a significant effect of time on the threshold for dendritic spike generation (L2/3: F17 = 43, L5: F8 = 11.91, L6: F6 = 10.08, p<0.001 for all layers)

To compare the time windows for somato-dendritic coupling between the different pyramidal cell classes we normalized the values at the different Δt's to the threshold for generating a dendritic spike without an axonal AP (Fig. 2A). 2-way repeated measurement ANOVA revealed that there was a significant effect of time (F_17_ = 27.17, p<0.001), no significant effect of layers (F_2_ = 1.13, p = 0.33) but a significant effect of the interaction between layers and time (F_34_ = 6.90, p<0.001). Post hoc test showed that the threshold reduction was significantly different for L5 pyramidal neurons compared to L6 and L2/3 for many time points, whereas L6 and L2/3 pyramidal neurons were only different from each other at one time point. The presence of an AP had the greatest effect on L5 pyramidal neurons reducing the threshold by 41±7%. Furthermore, the coincidence detection time window for L5 was extended relative to L6 and L2/3 pyramidal neurons (Fig. 2B).

## Discussion

In summary, we found that the coincidence timing curve for the initiation of dendritic spikes in L5 pyramidal neurons was wider than for L6 and for L2/3 pyramidal neurons. L6 and L2/3 pyramidal neurons exhibited similar coincidence detection windows to each other but were narrower than in L5 cells implying these cells require more precise synaptic inputs for this effect. The bAP had the greatest relative effect on dendritic spike generation in L5 neurons however the baseline threshold in L5 neurons was much larger than in L2/3 and L6 neurons (Table 1). Thus, the absolute dendritic spike threshold following a bAP was similar in all types of pyramidal neurons.

What are the implications of timing differences between pyramidal cell classes? We predict that processes in the dendritic tree which are influenced by the coupling of bAPs with local dendritic membrane potential such as STDP [25], [26], [27], [28], [29], local intrinsic excitability [30], [31], and release of retrograde messengers [32] will follow similar timing rules to those shown here. This has already been shown in the case of STDP in L5 pyramidal neurons where the STDP timing corresponds to the time window for dendritic spike generation and is reversed [29], [33] relative to the normal STDP time window in other neurons or for proximal inputs in pyramidal neurons [34], [35], [36], [37], [38], [39].

The active and passive properties of L6, L2/3 and L5 pyramidal tuft dendrites are similar but not identical [9], [10], [11], [12], [14], [40], [41]. This presumably also explains why the timing of BAC firing is different from cell type to cell type. The fact that there is a negative component to the time window for L5 cells, for instance, might reflect the influence of EPSPs on back-propagating APs which has been observed in these neurons before [20], [42]. Most importantly, when compared to L5 pyramidal neurons the amplitude and duration of the distal dendritic spike is reduced in L6 [10] and even more so in L2/3 neurons [19]. Under our conditions in vitro, L6 and L2/3 neurons therefore do not display bursts of axonal action potentials in response to an apical dendritic spike unlike the stereotypical bursting behaviour of L5 pyramidal neurons [21]. However, L2/3 pyramids have been shown to burst in vivo in the awake but not the anesthetized state [43], [44]. Along the same lines, dendritic activity has been shown to be greatly elevated in L5 neurons in awake versus anesthetized rats [45], [46]. This is consistent with the hypothesis that the awake state leads to an overall increase in dendritic excitability and shapes the output firing pattern in all pyramidal cell classes. The coupling of bAPs with dendritic input might therefore be even more crucial under physiologically relevant conditions.

The functional consequence of coincidence detection in pyramidal neurons depends also on the particular inputs that are associated. The cortical layer of the cell bodies and basal dendrites of pyramidal neurons determines the proximal input [47] and therefore determines the timing of APs propagating back into the tuft dendrite. The tuft dendrites of L2/3 and L5 both reach in to the uppermost layer of the cortex (L1) whereas L6 pyramidal neurons receive tuft input from upper L5 and L4. Since L1 receives long-range cortico-cortical feedback input, it has been suggested that L2/3 and L5 neurons can associate this input with the feed-forward and recurrent input in lower layers [18], [48], [49]. The cortex is also in constant dialogue with the thalamus via projections from L5 and L6 neurons and reciprocal connections from the thalamus to L4 and L1 [50], [51], [52]. Determining the functional implications of somato-dendritic coupling therefore awaits more precise data about the connectivity and timing of inputs to the different cortical layers under physiologically relevant conditions.

In conclusion, we have shown that all pyramidal neurons of the rat somatosensory cortex can associate inputs arriving at their distal and proximal dendritic trees in a limited time window that varies between cell classes. This suggests that pyramidal neurons operate in a similar way on the input which reaches the different cortical layers they are covering.
